# Treatment of cancer patients during the COVID-19 pandemic in the Philippines

**DOI:** 10.3332/ecancer.2020.1040

**Published:** 2020-05-08

**Authors:** Frederic Ivan Ting, Danielle Benedict Sacdalan, Honey Sarita Abarquez, Arnold John Uson

**Affiliations:** 1 University of the Philippines—Philippine General Hospital, Manila, 1000, Philippines; 2 Davao Doctors Hospital, Davao City, 8000, Philippines; 3 Perpetual Succour Hospital, Cebu City, 6000, Philippines; a http://orcid.org/0000-0002-9161-4918; b http://orcid.org/0000-0002-7400-8078; c http://orcid.org/0000-0001-5541-7389; d http://orcid.org/0000-0002-1262-9178

**Keywords:** treatment guideline, cancer patients, COVID-19 pandemic, Philippines

## Abstract

The COVID-19 pandemic has caused disruptions in cancer care around the world due to logistical and psychosocial reasons. This paper was written with the primary objective of providing a guide for medical oncologists in addressing concerns in the management of adult patients with solid tumours in the Philippines and for those working under similar circumstances. These recommendations are divided into prioritisation of cancer care, ensuring a safe work environment, organising the transition of cancer care, and maintaining cohesion in a time of isolation.

The coronavirus disease (COVID-19) is caused by a novel strain of coronavirus (SARS-CoV-2) that was first reported in Wuhan, China, in December 2019. On 30 January 2020, the Philippine Department of Health (DOH) reported the first case of COVID-19 in the country. Following a period of relative calm, on 7 March 2020, the first local transmission of COVID-19 was confirmed [1]. On 16 March 2020, the Philippine president Rodrigo Duterte placed the entire Luzon island under a state of enhanced community quarantine (ECQ). Under the ECQ, travel has been restricted for most people except for front-line medical, security personnel and persons delivering essential goods and services [2]. All establishments were closed except for the important services, such as banks and establishments that sell food and medicines with limited hours of service. This ECQ has also been further expanded to the different regions in the Visayas and Mindanao.

Previous data from China showed that older adults and the immunocompromised, such as cancer patients, are at higher risk of infection from SARS-CoV-2 and have a higher risk of developing severe complications. This risk may be attributable to the state of the patients’ underlying malignancy, to the treatment, or both [3–6].

Since the declaration of COVID-19 as a pandemic by the World Health Organization on 11 March 2020, various oncologic organisations, such as the American Society of Clinical Oncology (ASCO) [7] and the European Society of Medical Oncology (ESMO) [8], have issued several guidelines and recommendations to clinicians on the care of cancer patients during this pandemic.

Likewise, the Philippine Society of Medical Oncology crafted this paper to provide a guide for oncologists in addressing concerns in the management of adult cancer patients in the Philippines. These recommendations are divided into prioritisation of cancer care, ensuring a safe work environment, organising the transition of cancer care, and maintaining cohesion in a time of isolation.

## Prioritising cancer care

Oncologists are advised to prioritise cancer patients to be seen in the clinic or cancer centre based on the patients’ current underlying cancer status and their risk for COVID-19 infection. Several factors are to be considered, such as the patients’ cancer stage and tumour grade, tumour characteristics and tumour burden [9–13]. Based on individualised patient assessment, the decision of the attending oncologist on the need for immediate oncologic intervention classifies patients into those with *urgent* and* non-urgent* conditions.

Examples of cancer patients with **urgent conditions** are those with early or locally advanced cancer who have not completed their treatment and are eligible for surgery, adjuvant chemotherapy, radiation therapy, hormonal and biological therapy. Patients with advanced disease with large tumour burden who are currently symptomatic and those with oncologic emergencies are likewise included. These patients may warrant visits to the clinic and might need oncologic intervention because delaying treatment would decrease their survival but taking into consideration that the decision to advise the patient to come to the hospital should be balanced with the risk of the patient contracting the COVID-19 infection.

On the other hand, patients with stable disease, who are asymptomatic and have already completed their cancer treatment, are classified as those with **non-urgent conditions** that may warrant a less aggressive approach and clinic visits may be delayed.

The management of cases should be individualised with consideration to the tumour biology in terms of its growth characteristics, benefit of treatment, institutional resources (availability of treatment options in the facility), logistical concerns and regional differences (COVID-19 infection rate per locality or region) and the risks of infection.

The option to treat or delay treatment is best discussed with the patient within the context of multidisciplinary care. Shared decision making where the advantages of proceeding with treatment are weighed against the risks of delaying it and constraints imposed by the prevalence of COVID-19 infections. The final decision should be made after a thorough discussion with the patient and informed consent.

Oncologists may consider switching maintenance intravenous chemotherapy to oral regimens if such options are available. Likewise, switching to treatment schedules that require fewer clinic visits (e.g., extending to every 2–3 weeks instead of weekly infusion) has to be considered if possible. This is suggested primarily to safeguard the patients’ health by limiting their exposure to the clinic and to minimise the number of patients in the chemotherapy infusion unit in order to better comply with physical distancing recommendations.

Lastly, patients who have completed their treatment and are on surveillance should be encouraged to shift to technology platforms that allow remote consultations, such as telemedicine or phone calls, to facilitate continuation of care. DOH has come out with guidelines governing the use of telemedicine and electronic prescriptions in order to allow continuity of care while at the same time safeguarding data privacy [14–16]. Table 1 shows a summary of these recommendations.

Admittedly, some oncologists may have concerns regarding delays in treatment, but in the context of a health emergency treatment delays maybe a reasonable option to limit treatment visits, for the safety of the patient and to address logistical concerns.

## Ensuring a safe work environment

Oncology clinics and cancer institutions in the Philippines should remain COVID-19-free sanctuaries. Several precautions have to be in place to ensure the safety of both patients and healthcare providers (Table 2).

Patients can be screened remotely (e.g., over the phone for symptoms of COVID-19 infection prior to their scheduled visit) and promptly advised to seek appropriate care if symptoms are present in accordance with national health department guidelines [15]. A triage/screening area manned by personnel with appropriate personal protective equipment (PPE) should be in place in all cancer institutions to ensure the safety of both the patients and the healthcare worker.

Patients who are suspected of having signs or symptoms of COVID-19 need to be properly isolated from the rest of the patients, quarantined and referred to the appropriate specialist for proper management. Limiting the number of companions each patient can bring with them into the treatment facility is another important intervention to reduce the risk of COVID-19 in the oncology clinic.

Modifications to the physical plant and management of clinic facilities will also be necessary in order to control the flow of foot traffic. This will include limiting entry/exit to designated points. The areas where people naturally congregate, such as waiting and dining areas, may need to be actively monitored by clinic staff in order to strictly enforce physical distancing measures.

Vigorous infection and environmental control measures must be strongly implemented within the clinic. Clinic staff must be provided with appropriate PPE. Proper handwashing must always be strictly observed [17].

## Organising the transition of cancer care

**Figure 2.**
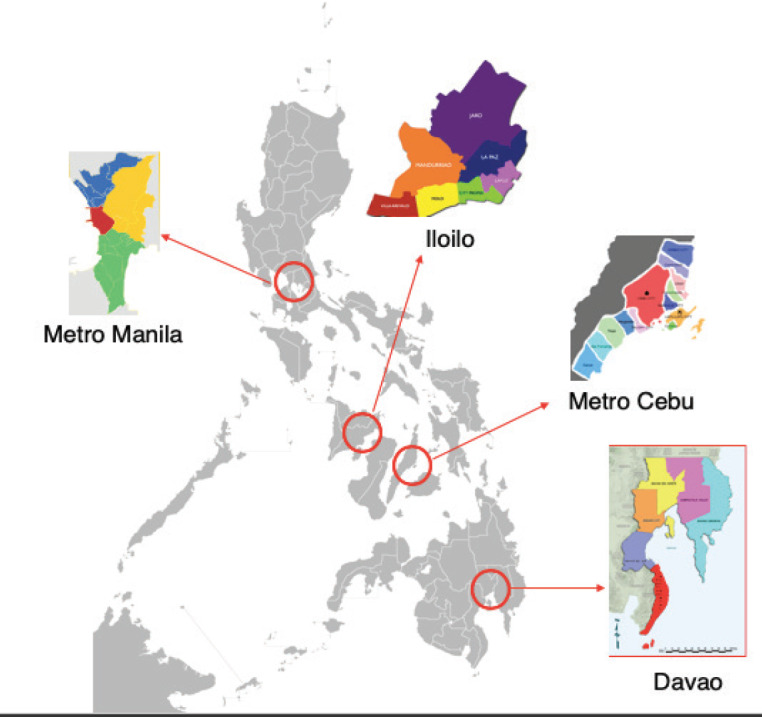
Map of the Philippine Islands highlighting the concentration of cancer treatment centres in the highly urbanized areas of Metro Manila, Metro Cebu, Iloilo City, and Davao City.

In the Philippines, cancer treatment centres are concentrated in highly urbanised areas, such as Metro Manila, Metro Cebu, Iloilo City and Davao City (Figure 2). Because of the archipelagic structure of the country, the current ECQ has made access to cancer care more difficult for patients due to the unavailability of public transportation and limitation of drug availability in the provinces. It is, therefore, of utmost importance that oncologists maintain communication with their cancer patients, whether through SMS or online communication platforms, such as email or social media, to discuss their medical concerns and treatment options. A key challenge during this time is uncertainty and the anxiety that arises from it. Open lines of communication with their physician will help to allay patient’s fears and avoid feelings of abandonment. Whenever possible, the help of mental health professionals may need to be enlisted in order to properly address patients’ concerns.

Some tertiary centres with high volume cancer centres have been designated as COVID-19 referral centres by the National Government. This new mandate requires these hospitals to temporarily cease or significantly limit services that are not essential to the management of COVID-19 patients. As a result, a large population of cancer patients are left without a place from which to receive care.

During this time oncologists working at these COVID-19 referral centres will need to refer many of their patients to other centres that are capable of continuing the patients’ cancer treatment if needed. Furthermore, cancer centres located in non-COVID-19 referral centres should assume care of these patients during this time of emergency. Despite these challenges, oncologists continue treating patients via the multidisciplinary approach and offer the best possible options after consideration of the available resources.

## Maintaining cohesion in a time of isolation

The challenges of community quarantine measures extend beyond the relationship between patients and oncologists. It also strains the connectivity between oncologists, who under different circumstances extensively collaborate in clinical and educational endeavours. It is under these circumstances that the importance of a national oncologist’s organisation comes to the fore. Through the centralised efforts of the national organisation, communication is maintained between the fragmented pockets of care that continue to function. In addition, evolving recommendations to guide treatment of patients are quickly disseminated to all oncologists in the country. This task is made easier by technology platforms that allow webinars and recorded messages to be prepared and communicated to the national membership of the organisation.

What was once the role of annual face-to-face scientific conferences is now being filled by teleconferencing and even in this realm national oncology societies can continue to play a central, anchoring role.

## Conclusion

This pandemic has forced us to rethink the way we approach the care of our patients. The speed with which the pandemic has spread has afforded us little time to understand how best to care for our cancer patients in this time. Certainly, our patients are particularly vulnerable to the effects of the medical and psycho-social effects of the current health emergency.

In the weeks and months ahead, more will be said and written for certain, but for now, and especially in places of the world like ours, we brace ourselves and do our best to proceed with care and compassion, remembering always that the safety of our cancer patients and their family, and of the healthcare workers is our utmost priority.

## Conflicts of interest

The authors have no conflicts of interest to declare.

## Funding

This paper did not receive any funding.

## Figures and Tables

**Figure 1.**
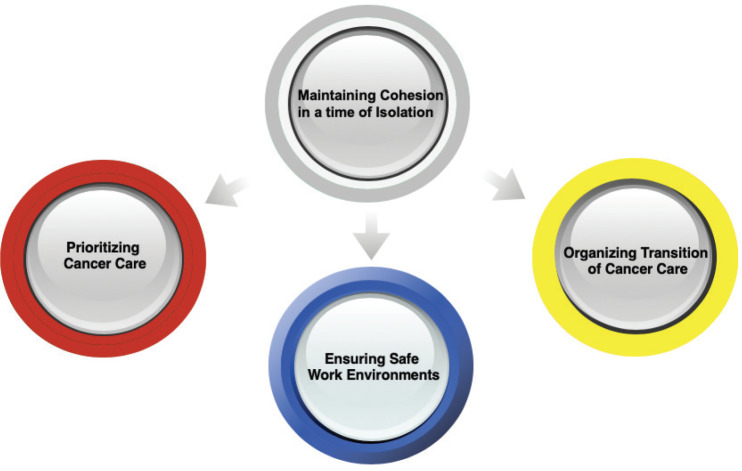
Recommendations are divided into prioritisation of cancer care, ensuring a safe work environment, organising the transition of cancer care, and maintaining cohesion in a time of isolation.

**Table 1.** Prioritisation of patients to be seen at the clinic.

Patients needing urgent treatment^1^ Treatment should be individualized (Logistical concerns, regional and institutional differences) Multidisciplinary discussion and shared decision making Consider treatment modification Consider telemedicine, communication with SMS, phone calls or email Patients on non-urgent treatment^2^ Consider treatment break Consider switching to oral regimen if possible Consider extending treatment intervals Consider telemedicine, communication with SMS, phone calls or email Patients on surveillance Consult via telemedicine/phone call or email. Use of e-prescriptions

1 Urgent treatment includes patients on adjuvant cytotoxic regimens and palliative regimens for patients with high tumour burden and/or impending visceral crisis.

2 Non-urgent treatment includes patients who are stable, have low tumour burden, and does not need immediate cytotoxic chemotherapeutic treatment as deemed by the attending physician.

**Table 2.** Precautions to ensure a safe workplace.

Controlling foot traffic by limiting one entry/exit point Setting up a triage or screening system Strictly enforcing physical distancing Wearing of the appropriate personal protective equipment (PPE) Proper and frequent handwashing

## References

[1] COVID-19 in the Philippines. https://www.who.int/philippines/emergencies/covid-19-in-the-philippines.

[2] Community Quarantine Over the Entire Luzon and Further Guidelines for the Management of the Coronavirus Disease 2019 Situation.

[3] Groups at High Risk for Complications of 2019-nCOV. https://www.cdc.gov/coronavirus/2019-ncov/specific-groups/high-risk-complications.html.

[4] Liang W, Guan W, Chen R (2020). Cancer patients in SARS-CoV-2 infection: a nationwideanalysis in China. Lancet Oncol.

[5] Zhang L, Zhu F, Xie L (2020). Clinical characteristics of COVID-19-infected cancer patients: a retrospective case study in three hospitals within Wuhan, China. Ann Oncol.

[6] Desai A, Sachdeva S, Parekh T (2020). COVID-19 and cancer: lessons from a pooled meta-analysis. JCO Glob Oncol.

[7] ASCO Coronavirus Information. https://www.asco.org/asco-coronavirus-information/provider-practice-preparedness-covid-19.

[8] Covid-19 and Cancer. https://www.esmo.org/newsroom/covid-19-and-cancer/supporting-oncology-professionals.

[9] Lambertini M, Toss A, Passaro A (2020). Cancer care during the spread of coronavirus disease 2019 (COVID-19) in Italy: young oncologists’ perspective. ESMO Open.

[10] Petrelli F, Zaniboni A, Ghidini A (2019). Timing of adjuvant chemotherapy and survival in colorectal, gastric, and pancreatic cancer. A systematic review and meta-analysis. Cancers.

[11] Kupstas AR, Hoskin TL, Day CN (2019). Effect of surgery type on time to adjuvant chemotherapy and impact of delay on breast cancer survival: a national cancer database analysis. Ann Surg Oncol.

[12] Francesco C, Pettke A, Michele B (2020). Managing COVID-19 in the oncology clinic and avoiding the distraction effect. Ann Oncol.

[13] Meattini I, Franco P, Belgioia L (2020). Radiation therapy during the coronavirus disease 2019 (covid-19) pandemic in Italy: a view of the nation’s young oncologists. ESMO Open.

[14] Implementation of the use of Electronic Means of Prescription for Drugs for the Benefit of Individuals Vulnerable to COVID-19. https://www.fda.gov.ph/fda-circular-no-2020-007-guidelines-in-the-implementation-of-the-use-of-electronic-means-of-prescription-for-drugs-for-the-benefit-of-individuals-vulnerable-to-covid-19/.

[15] Guidelines on the Use of Telemedicine in COVID-19 Response.

[16] Interim guidelines on 2019-nCoV. https://www.doh.gov.ph/2019-nCov/interim-guidelines.

[17] World Health Organization (2014). Clean hands protect against infection.

